# Supplementary material for: Acquired cancer cell resistance to T cell bispecific antibodies and CAR T targeting HER2 through JAK2 down-modulation
Source: Nat Commun. 2021 Feb 23;12:1237. doi: 10.1038/s41467-021-21445-4 (PMC7902842; doi:10.1038/s41467-021-21445-4)
Supplement: Supplementary file 1 — Supplementary Information [file 41467_2021_21445_MOESM1_ESM.pdf]

Supplementary information

Acquired cancer cell resistance to T cell bispecific antibodies and CAR T targeting HER2 through JAK2 down-modulation

Enrique J. Arenas[1, 2, #], Alex Martínez-Sabadell[1, #], Irene Rius Ruiz[1,2], Macarena Román Alonso[1], Marta Escorihuela[1], Antonio Luque[1], Carlos Alberto Fajardo[3], Alena Gros[3], Christian Klein[4], Joaquín Arribas[1, 2, 5, 6, 7]

[1] Preclinical Research Program Vall d'Hebron Institute of Oncology (VHIO), Barcelona, 08035, Spain.

[2] Centro de Investigación Biomédica en Red de Cáncer, Monforte de Lemos, Madrid, 28029, Spain.

[3] Cancer Immunotherapy & Immunology CAIMI Program, VHIO, Barcelona, 08035, Spain.

[4] Roche Innovation Center Zurich, Roche Pharmaceutical Research and Early Development, Wagistrasse 18, 8952 Schlieren, Switzerland.

[5] Department of Biochemistry and Molecular Biology, Universitat Autónoma de Barcelona, Campus de la UAB, 08193, Bellaterra, Spain.

[6] Cancer Research Program, IMIM (Hospital del Mar Medical Research Institute), Barcelona, Spain.

[7] Institució Catalana de Recerca i Estudis Avançats (ICREA), 08010, Barcelona, Spain.

[#] These authors contributed equally

Corresponding Author:

Joaquín Arribas PhD

Vall d'Hebron Institute of Oncology (VHIO)

C/Natzaret, 115-117

Barcelona 08035, Spain

Phone: +34 93 274 6026

E-mail: jarribas@vhio.net

Supplementary Fig. 1: Co-culture assays to determine the activity of TCBs and CARs, expression of HER2 in parental BT474 cells and BT-R resistant cells and sensitivity to different antitumor treatments.

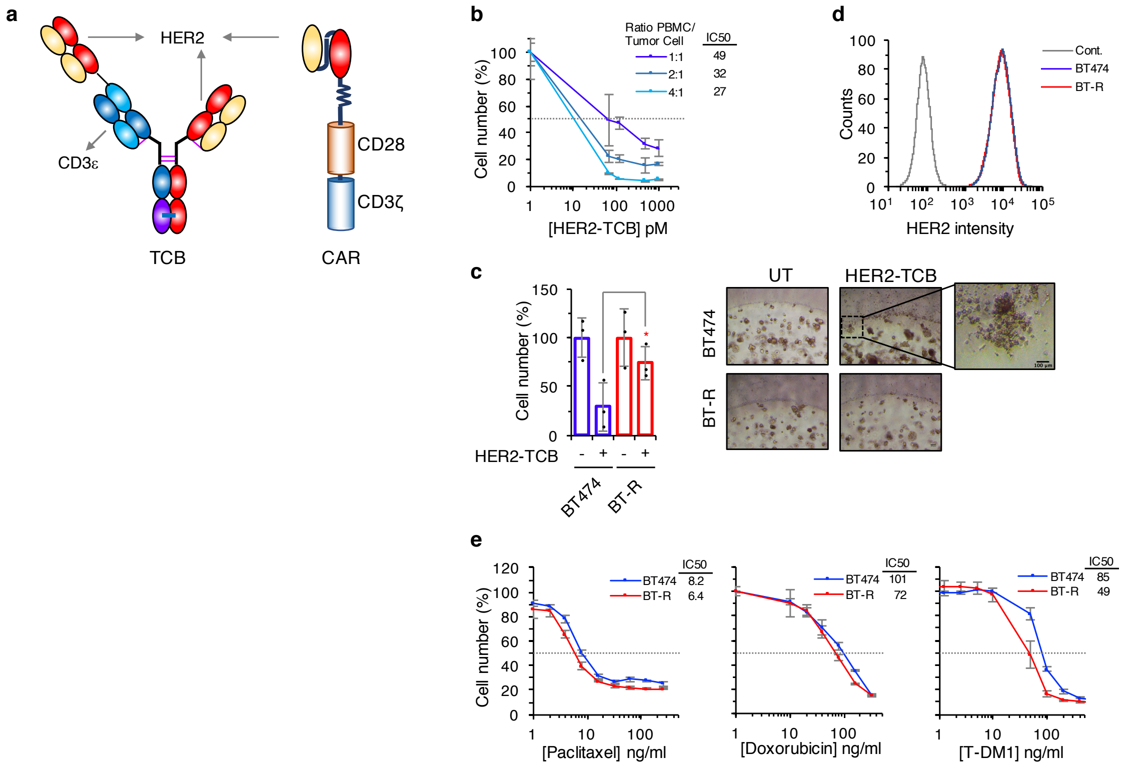

a, Schematic drawings of the HER2-TCB and HER2-CAR used in this study.

b, Co-cultures of PBMCs with BT474 cells at different ratios were treated with different concentrations of HER2-TCB for 72 h. Then, viable BT474 cells were quantified by flow cytometry using EpCAM as a marker.

c, Left, co-cultures of PBMCs with BT474 and resistant BT-R were grown in 3D and treated with 1 nM HER2-TCB for 72 h. Viable target cells were quantified as in **b** and normalized to untreated condition. *p=0.04, two-tailed t test.

Right, representative microphotographs of the 3D cultures.

d, Cells were stained with anti-HER2 and analyzed by flow cytometry. As control BT474 cells were stained with an irrelevant primary antibody.

e, Parental BT474 or resistant BT-R cells were treated with different concentrations of the indicated drugs. Treatment lasted 72 h in the chemotherapies treatments and 6 days in the T-DM1 experiment. Then, viable cells were quantified by the crystal violet assay.

Data are presented as mean ± SD of two (**b**) or three (**c**, **e**) independent experiments. Source data are provided as a Source Data file.

Supplementary Fig. 2: Cytokines and growth factors secreted and status of lymphocyte activation in co-cultures of parental BT474 cells and BT-R resistant cells.

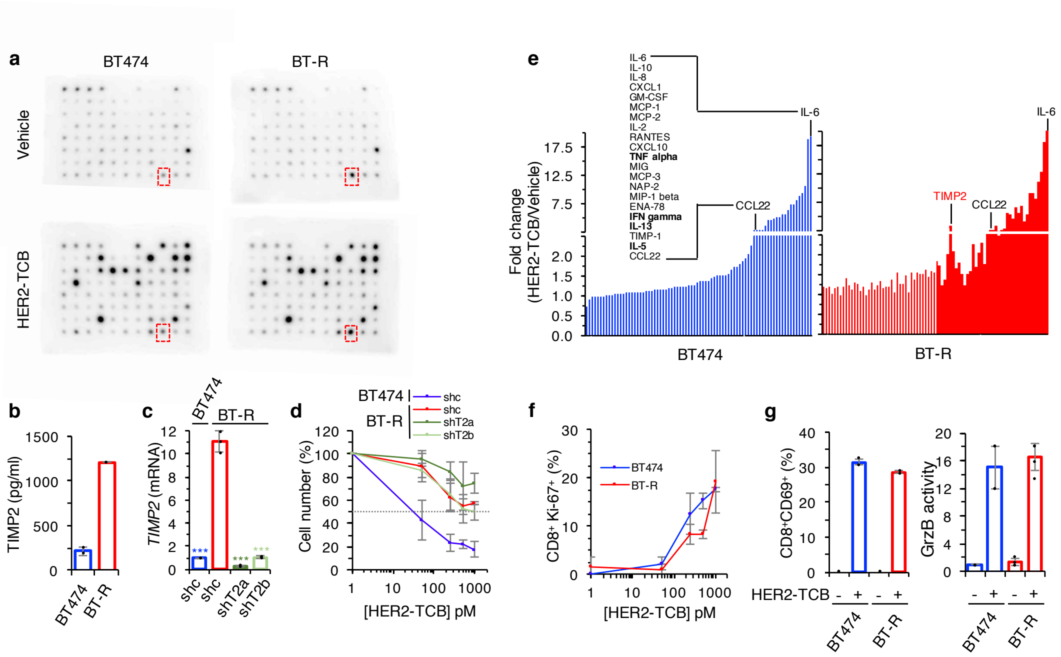

a, Analysis of a panel of cytokines and growth factors in the media conditioned by co-cultures of PBMCS with BT474 or BT-R cells treated with vehicle or HER2-TCB.

b, Expression of TIMP2 as determined by ELISA.

c, Expression of *TIMP2*, normalized to BT474, as determined by quantitative real-time PCR. ***p<0.001, two-tailed t test.

d, Co-cultures of PBMCs with the indicated cells were treated with different concentrations of HER2-TCB for 72 h. Then, viable target cells were quantified by flow cytometry using EpCAM as a marker.

e, Quantification of array showed in **a**. Data is shown as a fold change of co-cultures treated with HER2-TCB relative to co-cultures treated with vehicle. On the left and right, data on parental BT474 and BT-R, respectively.

f, The proliferation of lymphocytes was determined by Ki67 staining of $CD8^+$ cells in co-cultures treated with HER2-TCB for 72 h.

g, The percentage of $CD8^+CD69^+$ lymphocytes or Granzyme B activity were determined in co-cultures of PBMCs with parental BT474 or BT-R cells treated with 67.5 pM of the HER2-TCB for 72 h. $CD8^+CD69^+$ positivity was determined by flow cytometry. Granzyme B was measured by using the granzyme B substrate Ac-IEPD-pNA. Color reaction generated by the cleavage of the pNA substrate was measured. Results were normalized to untreated BT474 cells.

Data are presented as mean ± SD of two (**b**, **f**, **g** left), three (**c**, **d**), or four (**g** right) independent experiments. Source data are provided as a Source Data file.

Supplementary Fig. 3: IFN-γ signaling in cells knock-down or KO for IFNGR1 and knock-down of JAK1 and STAT1.

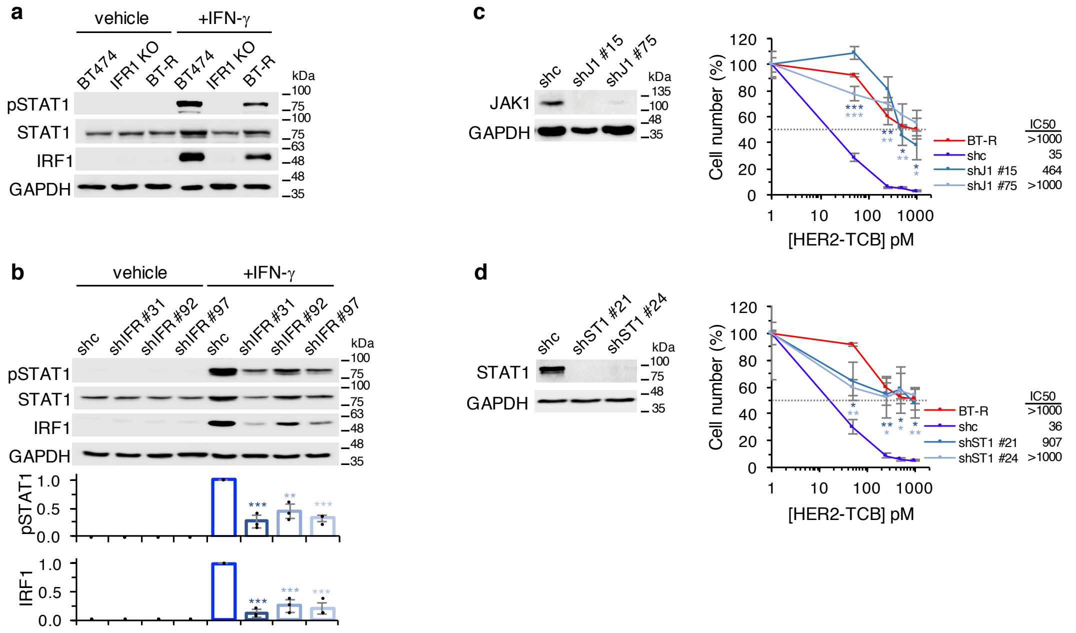

a, **b**, Western blot analysis of the expression of components of the IFN-γ intracellular signaling pathway components. Results were normalized to treated BT474 cells. **p=0.03, ***p<0.001, two-tailed t test.

c, d, Left, BT474 cells expressing control shRNA (shc) or shRNAs targeting JAK1 (shJ1) or STAT1 (shST1) were lysed and analyzed by Western blot with the indicated antibodies. Right, Co-cultures of PBMCs with BT-R cells or BT474 expressing the indicated shRNAs were treated with different concentrations of HER2-TCB for 72 h. Then, viable cells were quantified by flow cytometry using EpCAM as a marker. ***p<0.001, **p=0.005, *p=0.01, *p=0.03 (shJ1 #15), ***p<0.001, **p=0.002, **p=0.009, *p=0.01 (shJ1 #75); *p=0.03, **p=0.007, *p=0.01, *p=0.02 (shST1 #21); **p=0.003, *p=0.04, *p=0.04, **p=0.0042 (shST1 #24), two-tailed t test.

Data are presented as mean ± SD of three independent experiments. Source data are provided as a Source Data file.

Supplementary Fig.4: Role of IFN-γ signaling in the response of different HER2-positive cancer models to HER2-TCB.

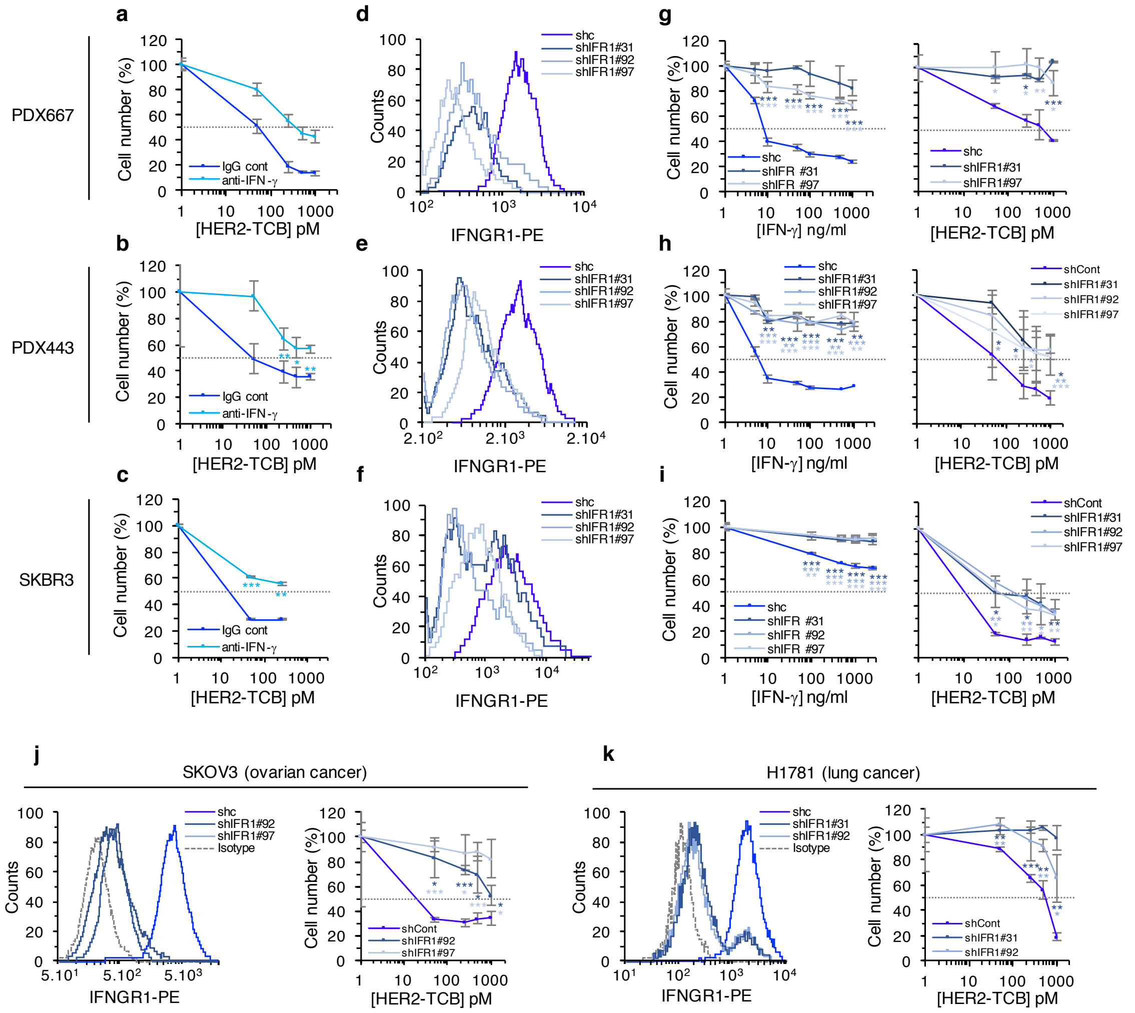

a, **b**, **c**, Co-cultures of PBMCs with the indicated breast cancer-derived cells were treated with different concentrations of HER2-TCB in presence of an IgG control or an IFN-γ blocking antibody for 72 h. Then, viable target cells were quantified by flow cytometry using EpCAM as a marker. Results are expressed as averages ± SD of two, four and three independent experiments, respectively.

d, **e**, **f**, Cells were stained with anti-IFNGR1 and analyzed by flow cytometry.

g, **h**, **i**, Left, the indicated cells were treated with different concentrations of IFN-γ for 5 days. Cell numbers were estimated with the crystal violet staining assay. The results of three independent experiments are expressed as averages ± SD.

Right, the indicated cells were treated with different concentrations of HER2-TCB and analyzed as in **a**, **b**, **c**.

j, k, Left, cells were analyzed as in **d**, **e**, **f**.

Right, cultures from the cell lines SKOV3 and H1781 derived from ovary and lung cancers respectively, were treated and analyzed as in **g**, **h**, **i**. Results are expressed as averages ± SD of three independent experiments.

(**b**) **p=0.008, *p=0.018, **p=0.007. (**c**) ***p<0.001, **p=0.002. (**g**) left, ***p<0.001; right, *p=0.04, ***p<0.001 (shIFNGR1 #31); *p=0.049, *p=0.013. **p=0.003, *p=0.012 (shIFNGR1 #97). (**h**) left, **p=0.002, ***p<0.001, **p=0.002 (shIFNGR1 #31); ***p<0.001, **p=0.002, **p=0.001 (shIFNGR1 #92); ***p<0.001, **p=0.007 (shIFNR1 #97); right, *p=0.02, *p=0.046, *p=0.01 (shIFNGR1 #31); *p=0.04, *p=0.014, **p=0.003 (shIFNGR1 #92); *p=0.02, *p=0.02, ***p<0.001 (shIFNGR1 #97). (**i**) left, **p=0.002, ***p<0.001; right, *p=0.04, *p=0.04, **p=0.005 (shIFNGR1 #31); **p=0.004, **p=0.005, *p=0.02 (shIFNGR1 #92); *p=0.012, **p=0.008, *p=0.013, **p=0.0014 (shIFNGR1 #97). (**j**) *p=0.03, ***p<0.001, *p=0.04, *p=0.04 (shIFNGR1 #92); ***p<0.001, *p=0.02, *p=0.02 (shIFNGR1 #97). (**k**) **p=0.005, ***p<0.001, **p=0.004, **p=0.002 (shIFNGR1 #31); **p=0.008, **p=0.007, *p=0.046 (shIFNGR1 #92). Two-tailed t test. Source data are provided as a Source Data file.

Supplementary Fig. 5: Resistance stability of BT-R cells and IFN-γ signaling in BT-R, BT474 or BT-RG cells engineered to gain or silence the expression of JAK2.

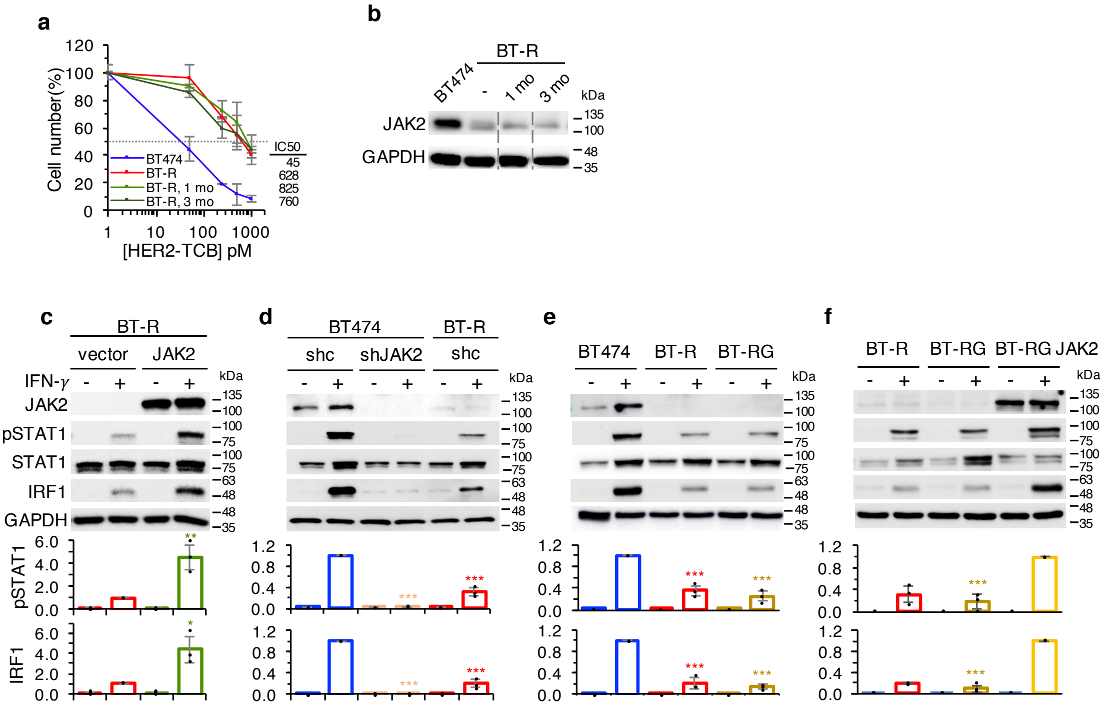

a, Co-cultures of PBMCs with parental BT474 or resistant BT-R cells kept under selective pressure or cultured in normal medium during 1 or 3 months were treated with different concentrations of HER2-TCB for 72 h. Then, viable cells were quantified by flow cytometry using EpCAM as a marker.

b, Levels of JAK2 as determined by Western blot on the same cells as in **a**.

c-f, Western blot analysis of the expression of components of the IFN-γ intracellular signaling pathway components.

(**c**) **p=0.009, *p=0.02. (**d**, **e**, **f**) ***p<0.001, two-tailed t test. Data are presented as mean ± SD of two (**a**) or three (**c**, **d**, **e**, **f**) independent experiments. Source data are provided as a Source Data file.

Supplementary Fig. 6: Generation of additional models of resistance to HER2-TCB in vivo.

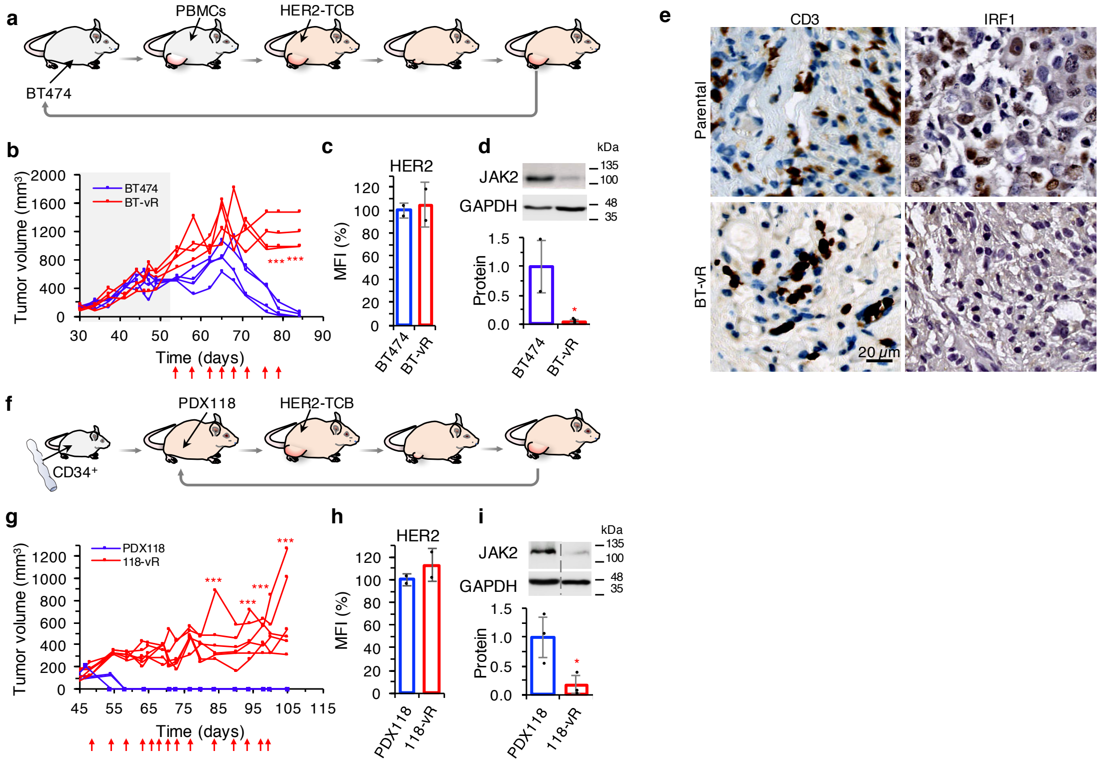

a, Schematic showing the generation of additional models. 10^7 BT474 cells were injected orthotopically into NSG mice. When tumors reached ~200 mm^3, 10^7 PBMCs were injected i.p. Then animals were treated i.v. with an increasing concentration of HER2-TCB. Tumors were allowed to regrow, excised, and injected again into NSG mice.

b, Mice were injected with 10^7 BT474 or BT-vR cells and treated with 0.25 mg/kg HER2-TCB. Treatments are indicated by red arrows. Tumor volumes of individual mice are represented (n=4 per group).

c, Cells were stained with anti-HER2 and analyzed by flow cytometry. Quantitative data, normalized to BT474, corresponds to averages ± SD of two determinations.

d, Levels of JAK2 as determined by Western blot. Quantitative data, normalized to BT474, corresponds to averages ± SD of two different parental and four different resistant tumor determinations.

e, Representative immunohistochemical staining of hCD3 and hIRF1 in tumor sections. Images are representative of all tumors in **b**.

f, Schematic showing the generation of additional models. PDX118 was grafted in mice humanized with CD34$^+$ cells. When tumors reached ~200 mm^3, animals were treated i.v. with an increasing concentration of HER2-TCB. Tumors were allowed to regrow and treatment was repeated.

g, Mice humanized with CD34$^+$ cells were grafted with PDX118, or 118-vR tumors. Mice were treated i.v. with 0.25 mg/kg HER2-TCB. Tumor volumes of individual mice are represented (PDX118, n=3; 118-vR, n=6).

h, **i**, Levels of HER2 or JAK2 were determined as in **c**, **d**. Quantitative data corresponds to averages ± SD of two (**h**) and three (**i**) determinations.

(**d**) *p=0.02. (**i**) *p=0.04, two-tailed t test. (**b** and **g**) ***p<0.001, two-way ANOVA and Bonferroni correction. Source data are provided as a Source Data file.

Supplementary Fig. 7: Models of resistance to IFN-γ are also resistant to HER2-TCB because of JAK2 downmodulation.

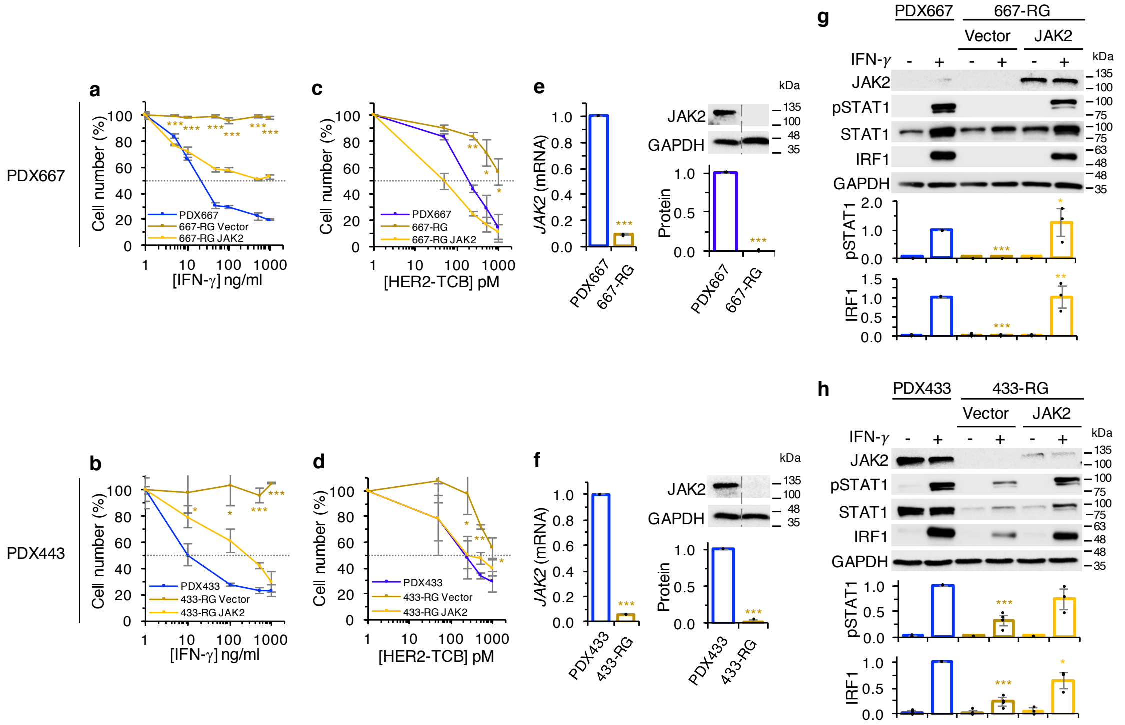

a, **b**, The indicated cells were treated with different concentrations of IFN-γ for 5 days. Cell numbers were estimated with the crystal violet staining assay.

c, **d**, Co-cultures of the indicated cells were treated with different concentrations of HER2-TCB for 72 h. Then, viable cells were quantified by flow cytometry using EpCAM as a marker.

e, **f**, The levels of JAK2 (mRNA and protein) as determined by quantitative real-time PCR (left) or Western blot (right). Results were normalized to parental cells.

g, **h**, Western blot analysis of the expression of components of the IFN-γ intracellular signaling pathway components. Results were normalized to treated parental cells.

(**a**) ***p<0.001. (**c**) **p= 0.004, *p=0.012, p*=0.02. (**e**) ***p<0.001. (**g**) *p=0.02, **p=0.008, ***p<0.001. (**b**) *p=0.018, *p=0.014, ***p<0.001. (**d**) *p=0.02, **p=0.002, *p=0.013. (**f**) ***p<0.001. (**h**) *p=0.04, ***p<0.001, two-tailed t test. Data are presented as mean ± SD of three independent experiments. Source data are provided as a Source Data file.

Supplementary Fig. 8: Gating strategies used for functional assays, protein expression and cell sorting.

Gating strategy I

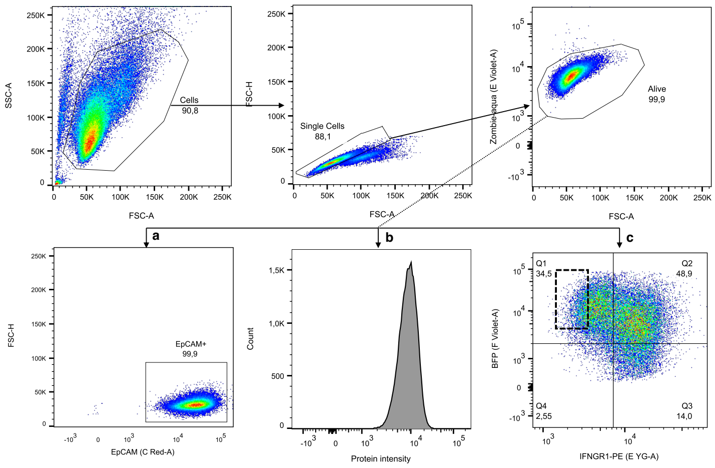

Gating strategy II

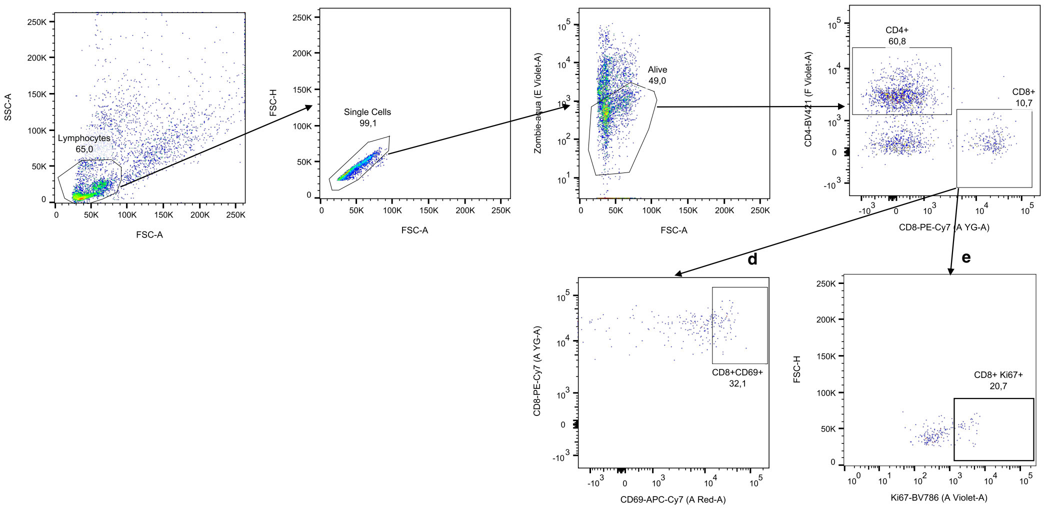

a, Gating strategy to obtain EpCAM^{+} cell counts in functional T cell cytotoxicity assays presented on **Fig. 1b,1f, Fig. 3a, b,g,h, Fig. 5b,c,g,h, Fig. 6b,c,g,h, Supplementary Fig. 1b,c, Supplementary Fig. 2d, Supplementary Fig. 3c,d, Supplementary Fig. 4a-c,g-k, Supplementary Fig. 5a, Supplementary Fig 7c,d**.

b, Gating strategy to measure MFI for a particular surface protein: HER2 (presented on **Supplementary Fig. 1d, Supplementary Fig. 6c**,**h**), HER2-TCB binding (presented on **Fig 1j**), IFNGR1 (presented on **Fig. 3e, Fig. 4c, Supplementary Fig. 4d-f**,**j**,**k**), IFNGR2 (presented on **Fig. 4c**), PDL1, PDL2, CD80, CD86, Galectin-9, B7-H3, B7-H4, HVEM, ICOS-L, 41BB-L, OX40-L (presented on **Fig. 1k**). Same strategy was used for Annexin V^{+} analysis presented on **Fig. 3d**, but in this case the viability marker used was PI.

c, Gating strategy to sort BFP^{high}/IFNGR1 (square dashes) negative expressing cells from BT474 cells expressing Cas9 and a CRISPR gRNA targeting IFNGR1, used for in vitro cytotoxic assays (presented on **Fig. 3f-h**) and in vivo (presented on **Fig. 3i**).

d, e, Gating strategy to obtain the % of CD69^{+} (**d**) and Ki67^{+} cells (**e**) from CD8^{+} cells used in functional in vitro T cell cytotoxicity assays, presented on **Supplementary Fig. 2f** and **g**.
