# Supplementary material for: Acquired cancer cell resistance to T cell bispecific antibodies and CAR T targeting HER2 through JAK2 down-modulation
Source: Nat Commun. 2021 Feb 23;12:1237. doi: 10.1038/s41467-021-21445-4 (PMC7902842; doi:10.1038/s41467-021-21445-4)
Supplement: Supplementary file 3 — Reporting Summary [file 41467_2021_21445_MOESM3_ESM.pdf]

nature research

Corresponding author(s): Joaquín Arribas

Last updated by author(s): Jan 11, 2021

Reporting Summary

Nature Research wishes to improve the reproducibility of the work that we publish. This form provides structure for consistency and transparency in reporting. For further information on Nature Research policies, see our Editorial Policies and the Editorial Policy Checklist.

Statistics

For all statistical analyses, confirm that the following items are present in the figure legend, table legend, main text, or Methods section.

n/a	Confirmed	
☐	☒	The exact sample size (*n*) for each experimental group/condition, given as a discrete number and unit of measurement
☐	☒	A statement on whether measurements were taken from distinct samples or whether the same sample was measured repeatedly
☐	☒	The statistical test(s) used AND whether they are one- or two-sided<br>*Only common tests should be described solely by name; describe more complex techniques in the Methods section.*
☒	☐	A description of all covariates tested
☒	☐	A description of any assumptions or corrections, such as tests of normality and adjustment for multiple comparisons
☐	☒	A full description of the statistical parameters including central tendency (e.g. means) or other basic estimates (e.g. regression coefficient) AND variation (e.g. standard deviation) or associated estimates of uncertainty (e.g. confidence intervals)
☐	☒	For null hypothesis testing, the test statistic (e.g. *F*, *t*, *r*) with confidence intervals, effect sizes, degrees of freedom and *P* value noted<br>*Give P values as exact values whenever suitable.*
☒	☐	For Bayesian analysis, information on the choice of priors and Markov chain Monte Carlo settings
☒	☐	For hierarchical and complex designs, identification of the appropriate level for tests and full reporting of outcomes
☒	☐	Estimates of effect sizes (e.g. Cohen's *d*, Pearson's *r*), indicating how they were calculated

Our web collection on statistics for biologists contains articles on many of the points above.

Software and code

Policy information about availability of computer code

Data collection	BD FACSDiva v 6.1.2 software, SDS 2.4 software
Data analysis	FlowJo v10 software, NDP.view2.7 software, Fiji v1.48p, ImageJ v1.49b, R.Q manager v1.2 software, DataAssist v3.01 software, software Real Time Analysis (RTA v2.7.7), STAR/2.5.3a, RSEM/1.3.0, GEMtools v1.7.0, DESeq2/1.18, fgsea R package v1.16.0, pheatmap R package v1.0.12, GSEAPreranked v6.0.12, R v3.6.3 software, GraphPad Prism v6.0c.

For manuscripts utilizing custom algorithms or software that are central to the research but not yet described in published literature, software must be made available to editors and reviewers. We strongly encourage code deposition in a community repository (e.g. GitHub). See the Nature Research guidelines for submitting code & software for further information.

Data

Policy information about availability of data

All manuscripts must include a data availability statement. This statement should provide the following information, where applicable:

- Accession codes, unique identifiers, or web links for publicly available datasets
- A list of figures that have associated raw data
- A description of any restrictions on data availability

RNAseq data that support the findings of the study have been deposited in Sequence Read Archive (SRA) and are accessible through the SRA Bioproject accession number PRJNA674313 (https://www.ncbi.nlm.nih.gov/bioproject/?term=PRJNA674313). All other relevant data are available from the corresponding author on request.

Field-specific reporting

Please select the one below that is the best fit for your research. If you are not sure, read the appropriate sections before making your selection.

☒ Life sciences ☐ Behavioural & social sciences ☐ Ecological, evolutionary & environmental sciences

For a reference copy of the document with all sections, see nature.com/documents/nr-reporting-summary-flat.pdf

Life sciences study design

All studies must disclose on these points even when the disclosure is negative.

Sample size	An assessment of the number of animals required for each procedure was performed using Statistical Power Analysis and taking into consideration the appropriate statistical tests, significance level of 5% and statistical power of 80%. An estimate of variance was inferred from previous experiments, especially considering the variability of tumor xenograft growth.<br>For in vitro experiments, two to four biological replicates were performed, which was sufficient to show data reproducibility. No sample-size calculations were performed.
Data exclusions	Animals were excluded from the study if presented graft vs host disease. Criteria was pre-established and animals were excluded from all humanized PBMCs experiments.
Replication	Experimental findings were reliably reproduced. Experiments were performed at least three times unless otherwise noted in the manuscript.
Randomization	Animals, upon arrival, were randomly allocated into cages with five mice each. The mice were randomly assigned to experimental groups. No specific method of randomization was used.<br>For in vitro experiments, randomization was not needed, since samples were not divided into groups.
Blinding	Experiments were performed in a blinded fashion.

Reporting for specific materials, systems and methods

We require information from authors about some types of materials, experimental systems and methods used in many studies. Here, indicate whether each material, system or method listed is relevant to your study. If you are not sure if a list item applies to your research, read the appropriate section before selecting a response.

Materials & experimental systems

n/a	Involved in the study	
☐	☒	Antibodies
☐	☒	Eukaryotic cell lines
☒	☐	Palaeontology and archaeology
☐	☒	Animals and other organisms
☐	☒	Human research participants
☒	☐	Clinical data
☒	☐	Dual use research of concern

Methods

n/a	Involved in the study	
☒	☐	ChIP-seq
☐	☒	Flow cytometry
☒	☐	MRI-based neuroimaging

Antibodies

Antibodies used

Anti-Human HER2 (clone CB11) BioGenex Cat# AM134
Anti-Human JAK1 (clone 6G4) Cell Signaling Technology Cat# 3344, RRID: AB_2265054
Anti-Human JAK2 (clone D2E12) Cell Signaling Technology Cat# 3230, RRID: AB_2128522
Anti-Human p-STAT1 (Tyr701) (clone 58D6) Cell Signaling Technology Cat# 9167, RRID:AB_561284
Anti-Human STAT1 Cell Signaling Technology Cat# 9172, RRID:AB_2198300
Anti-Human IRF1 (C-20) Santa Cruz Biotechnology Cat# sc-497, RRID:AB_631838
Anti-Human GAPDH [EPR6256] Abcam Cat# ab128915, RRID:AB_11143050
Sheep Anti-Mouse IgG - Horseradish Peroxidase antibody GE Healthcare Cat# NA931, RRID:AB_772210
Donkey Anti-Rabbit IgG - Horseradish Peroxidase antibody GE Healthcare Cat# NA934, RRID:AB_772206
Anti-Human IRF1 Atlas Antibodies/Sigma-Aldrich Cat# HPA063131, RRID:AB_2684945
EnVision+ System- HRP Labelled Polymer Anti-Rabbit Dako/Agilent Cat# K4003, RRID:AB_2630375
Anti-Human CD3 (clone 2GV6) Ventana Medical Systems Cat# 790-4341, RRID:AB_2335978
Trastuzumab (Herceptin) Roche
Goat anti-Human IgG (H+L) Cross-Adsorbed Secondary Antibody, Alexa Fluor 488 Invitrogen Cat# A-11013, RRID:AB_2534080
Anti-Human PD-L1 (clone 29E.2A3) Biolegend Cat# 329736, RRID:AB_2629582
Anti-Human PD-L2 (clone MIH18) Miltenyi Biotec Cat# 130-098-525, RRID:AB_2656873

	Anti-Biotin PE-Vio770 Miltenyi Biotec Cat# 130-113-292, RRID:AB_2726074<br>Anti-Human CD80 (clone 2D10) Biolegend Cat# 305221, RRID:AB_10899567<br>Anti-Human CD86 (clone IT2.2) Biolegend Cat# 305425, RRID:AB_10899582<br>Anti-Human Galectin-9 (clone 9M1-3) Biolegend Cat# 348905, RRID:AB_10613284<br>Anti-Human CD276 (B7-H3) (clone MIH42) Biolegend Cat# 351003, RRID:AB_10719959<br>Anti-Human B7-H4 (clone MIH43) Biolegend Cat# 358103, RRID:AB_2562080<br>Anti-Human CD270 (HVEM) (clone 122) Biolegend Cat# 318805, RRID:AB_2203704<br>Anti-Human CD275 (ICOS-L) (clone 2D3) Biolegend Cat# 309403, RRID:AB_314769<br>Anti-Human CD137L (41BB-L) (clone 5F4) Biolegend Cat# 311503, RRID:AB_314882<br>Anti-Human CD252 (OX40-L) (clone 11C3.1) Biolegend Cat# 326307, RRID:AB_2207272<br>Anti-Human CD119 (IFN-gamma R alpha chain) (clone GIR-208) Biolegend Cat# 308606, RRID:AB_314726<br>Anti-Human IFN-gamma R β chain (clone 2HUB-159) Biolegend Cat# 308504, RRID:AB_314718<br>Anti-Human CD326 (EpCAM) (clone 9C4) Biolegend Cat# 324212, RRID:AB_756086<br>Anti-human CD34 (clone 581) StemCell Technologies Cat# 60013, RRID:AB_2783003<br>Anti-Human CD45 (clone HI30) Biolegend Cat# 304008, RRID:AB_314396<br>Anti-Human CD3 Biolegend Cat# 300408, RRID:AB_314062<br>CD3 Monoclonal Antibody (Clone OKT3) Thermo-Fisher Cat# 16-0037-85, RRID:AB_468855<br>LEAF Purified anti-human IFN gamma antibody Biolegend Cat# 506512, RRID:AB_315445<br>LEAF purified IgG1 mouse control Biolegend Cat# 400123<br>Anti-Ki-67 (clone B5) BD Biosciences Cat# 563756, RRID:AB_2732007<br>Anti-Human CD8 (Clone SK1) Biolegend Cat# 344712, RRID:AB_2044008<br>Anti-Human CD69 (clone FN50) Biolegend Cat# 310914, RRID:AB_314849<br>PE Mouse IgG1, κ Isotype Ctrl (Clone MOPC-21) Biolegend Cat# 400114<br>Rabbit IgG Sigma-Aldrich Cat# I8140, RRID:AB_1163661<br>Anti-H3K27Me3 Millipore Cat# 07-449, RRID:AB_310624<br>Anti-H3K27Ac Abcam Cat# ab4729, RRID:AB_2118291
Validation	All antibodies used in this study were validated by the manufacturers for specific detection of the antigen and species reactivity.<br>Anti-Human HER2 was validated by IHC staining of human breast carcinoma tissue FFPE.<br>All CST primary antibodies were validated for WB in human samples.<br>Anti-Human IRF1 (SC) has been validated for WB in human samples in several publications such as PMID #8626684 and PMID #17148586.<br>Anti-Human GAPDH was validated for WB in human samples.<br>Anti-Human IRF1 (Atlas Antibodies/Sigma-Aldrich) was validated for IHC in human samples.<br>Anti-Human CD3 (Ventana) was validated for IHC in human samples.<br>All Biolegend primary antibodies were validated for FC in human samples.<br>Anti-Human PD-L2 was validated for FC in human samples.<br>Anti-human CD34 was validated for FC in human samples.<br>CD3 Monoclonal Antibody (Clone OKT3) was validated for FC in human samples.<br>Anti-Ki-67 was validated for FC in human samples.<br>Anti-H3K27Me3 was validated by WB in human samples.<br>Anti-H3K27Ac was validated for ChIP in human samples.

Eukaryotic cell lines

Policy information about cell lines

Cell line source(s)	BT474 (#HTB-20), SKBR3 (#HTB-30), HEK293T (#CRL-11268), SKOV3 (#HTB-77) and H1781 (#CRL-5894) were obtained from ATCC (Manassas, VA, USA). GP2-293 cells (#631458) were obtained from Clontech. PDX433, PDX667, and PDX118 come from patient samples from Vall d'Hebron Hospital and have been established at VHIO following institutional guidelines.
Authentication	Cell lines were purchased with the certificate from the vendor. Cell lines were not authenticated in-house. Patient samples were characterized in Vall d'Hebron Hospital for the presence of ER and HER2 by IHC.
Mycoplasma contamination	Cell lines were tested for mycoplasm and only mycoplasma-negative cell lines were used.
Commonly misidentified lines<br>(See ICLAC register)	None of the cell lines used in this study was found in the database of commonly misidentified cell lines that are maintained by ICLAC.

Animals and other organisms

Policy information about studies involving animals; ARRIVE guidelines recommended for reporting animal research

Laboratory animals	Mice, NOD.Cg-Prkdcscid Il2rgtm1Wjl/SzJ (NSG), female, 5 to 7 weeks of age. All mice in this study were kept within Home Office limits of 22ºC ± 2ºC, 55 – 65% humidity and run on a 12 h light/dark cycle that runs from 8am to 8pm.

Wild animals	This study did not involve wild animals.
Field-collected samples	This study did not involve samples collected from the field.
Ethics oversight	Animal work was performed according to protocols approved by the Ethical Committee for the Use of Experimental Animals at the Vall d'Hebron Institute of Oncology.

Note that full information on the approval of the study protocol must also be provided in the manuscript.

Human research participants

Policy information about [studies involving human research participants]

Population characteristics	Patient derived xenografts were obtained from HER2+ breast cancer patients.<br>PBMCs were obtained from healthy donors.
Recruitment	Patient derived xenografts were obtained from HER2+ breast cancer patients from Vall d'Hebron Hospital.<br>PBMCs were obtained through the Blood and Tissue Bank of Catalonia (BST) from healthy donors.
Ethics oversight	All human samples were obtained with informed consent and following institutional guidelines under protocols approved by the institutional review boards (IRBs) at Vall d'Hebron Hospital in accordance with the declaration of Helsinki.

Note that full information on the approval of the study protocol must also be provided in the manuscript.

Flow Cytometry

Plots

Confirm that:

- [x] The axis labels state the marker and fluorochrome used (e.g. CD4-FITC).
- [x] The axis scales are clearly visible. Include numbers along axes only for bottom left plot of group (a 'group' is an analysis of identical markers).
- [x] All plots are contour plots with outliers or pseudocolor plots.
- [x] A numerical value for number of cells or percentage (with statistics) is provided.

Methodology

Sample preparation	Cultured cells were collected by trypsinization. Cells from tumor samples were purified using a physical and enzymatic protocol. Briefly, tumours were digested in 300 U/ml collagenase IA and 100 U/ml Hialuronidase IS in DMEM F-12 medium. After 1 hour of incubation at 37°C with shaking at 80 rpm, the mixture was filtered through 100-micromter strainers. Red blood cells were lysed with 1x Red Blood Cell lysis buffer for 5 min RT. After a wash with 1x PBS, samples were acquired on LSR Fortessa.
Instrument	For cell sorting: BD FACSAria 1.0<br>For flow cytomerty analysis: BD LSRFortessa
Software	For data collection: BD FACSDiva v 6.1.2 software<br>For data analysis: FlowJo v 10
Cell population abundance	More than 99% of the population was negative for IFNGR1 after sorting the BT474 KO IFNGR1 cell line. Validation was done by IFNGR1 staining.
Gating strategy	After cells were selected in the FSC/SSC dot plot to remove debris, they were gated to exclude cellular aggregates in the FSC/FSC dot plot. To obtain EpCAM+ counts in functional T-cell cytotoxicity assays and surface marker expression, Zombie Aqua negative cells were further analyzed with the following antibodies: HER2, binding HER2-TCB, IFNGR1, IFNGR2, PDL1, PDL2, CD80, CD86, Galectin-9, B7-H3, B7-H4, HVEM, ICOS-L, 41BB-L, OX40-L. Same strategy was used for Annexin V+ analysis, but in this case the viability marker used was PI. Same gating strategy was used to sort BFPhigh/IFNGR1 negative expressing cells from BT474 cells expressing Cas9 and a CRISPR gRNA targeting IFNGR1.<br><br>In the case of obtaining the % of CD69+ and Ki67+ in CD8+, cells were selected in the FSC/SSC dot plot to remove debris and they were gated to exclude cellular aggregates in the FSC/FSC dot plot. Then, Zombie Aqua negative cells were gated using the CD4 and CD8 antibodies. Finally, CD8+ cells were gated for CD69 and Ki67 positivity.

- [x] Tick this box to confirm that a figure exemplifying the gating strategy is provided in the Supplementary Information.
